# Back From the Brink: Alterations in B and T Cell Responses Modulate Recovery of Rainbow Trout From Chronic Immunopathological *Tetracapsuloides bryosalmonae* Infection

**DOI:** 10.3389/fimmu.2020.01093

**Published:** 2020-06-03

**Authors:** Christyn Bailey, Helmut Segner, Thomas Wahli, Carolina Tafalla

**Affiliations:** ^1^Fish Immunology and Pathology Group, Animal Health Research Center (CISA-INIA), Madrid, Spain; ^2^Centre for Fish and Wildlife Health, University of Bern, Bern, Switzerland

**Keywords:** fish-host-parasite interactions, proliferative kidney disease (PKD), B cells, chronic immunopathology, recovery

## Abstract

Proliferative kidney disease (PKD) caused by the myxozoan parasite *Tetracapsuloides bryosalmonae* is one of the most serious infectious diseases negatively impacting farmed and wild salmonids throughout Europe and North America. PKD pathogenesis results in a massive B cell proliferation and dysregulation with aberrant immunoglobulin production and plasma cell differentiation along with a decrease in myeloid cells and inhibition of innate pathways. Despite the huge immunopathological reaction in the kidney during infection, under specific conditions, fish can survive and return to full fitness. Fish are unique in this ability to recover renal structure and functionality from extensive tissue damage in contrast to mammals. However, only limited knowledge exists regarding the host immune response coinciding with PKD recovery. Moreover, almost no studies of the immune response during disease recovery exist in fish. We utilized the rainbow trout–*T. bryosalmonae* system as an immunological model of disease recovery. Our results demonstrated that recovery is preceded by an intense immune response at the transcript level, decreasing parasite burden, and an increased degree of kidney inflammation. Later in the recovery phase, the immune response transpired with a significant decrease in lymphocytes and an increase in myeloid cells. These lymphocytes populations contained lower levels of B cells comparative to the control in the anterior and posterior kidney. Additionally, there was downregulation of several transcripts used as markers for plasma cells (*blimp1, igt sec, igm sec, igd sec*, and *cd38*) and T cell subsets (*cd4, cd8*α, *cd8*β, and *tcr*β). The decrease in these T cell transcripts significantly correlated with decreasing parasite intensity. Alternatively, there was strong upregulation of *pax-5* and *igt mem*. This suggests a change in B cell processes during the recovery phase relative to clinical PKD may be necessary for the host to re-establish homeostasis in terms of an arrest in the dominant antibody like response transitioning to a transcriptional profile associated with resting B cells. The knowledge generated here in combination with earlier studies illuminates the full power of analyzing the entire trajectory of disease from the normal healthy state to recovery enabling the measurement of an immune response to pinpoint a specific disease stage.

## Introduction

Proliferative kidney disease (PKD), one of the most serious emerging infectious diseases of salmonid fish, is caused by the myxozoan parasite *Tetracapsuloides bryosalmonae*. PKD is accountable for substantial economic losses in the aquaculture industry and is also responsible for hampering wild salmonid populations in Europe and North America (1, 2). The PKD-causing parasite, *T. bryosalmonae* has a two-host life cycle exploiting salmonid fish as an intermediate vertebrate host and freshwater bryozoa as an invertebrate host (3). *T. bryosalmonae* parasites in the form of malacospores are released from bryozoans infecting the fish host via the gills, eventually traveling via the blood to the main target organ, the posterior kidney, for development (4). Here, *T. bryosalmonae* penetrates the interstitial tissue, develops and differentiates from extrasporogonic to sporogonic stages and, provokes a chronic immunopathology characterized by a massive renal swelling, lymphocytic hyperplasia, and hyperimmunoglobulinemia (1, 4, 5). Eventually, fish malacospores infective to bryozoans are released with the urine (3). Histopathological alterations observed in the posterior kidney during PKD pathogenesis include a reduction in melanomacrophage centres, proliferative and granulomatous nephritis, necrotizing vasculitis with thrombus formation and a strong hyperplastic response and a systemic deterioration of renal tubules (4, 6). Parasites can also proliferate and cause a reaction in the anterior kidney, the spleen and the liver (4).

Much of the knowledge of the immune response during PKD pathogenesis originated from the model species rainbow trout (*Oncorhynchus mykiss*) exposed to the European strain of the parasite, for which this non-native trout species acts as a dead-end host. Understanding this host-parasite interaction is particularly important as intense aquaculture losses occur in this system in Europe (1). The two most notable cellular aspects of the host response are a decrease in myeloid cells, suggested to be an inhibition of the innate immune response caused by the parasite and the *in-situ* proliferation of lymphocytes in the anterior and posterior kidney (7, 8). Transcriptional findings have described a flood of cytokines involved in an imbalanced T-helper (Th)—like response and anti-inflammatory like processes, a dysregulated B cell antibody type reaction and a suppression of several pro inflammatory cytokines (8–10).

Recently, a characterization of the B cell dysregulation provoked by the parasite was undertaken at the protein and cellular level (11). Abos et al. (11) demonstrated that all fish immunoglobulin (Ig) isotypes (IgM, IgD, and IgT) were increased at protein level in the posterior kidney during PKD pathogenesis and that four different B cell subsets (IgM+IgD+, IgM+IgD–, IgD+IgM–, and IgT+ cells) coexisted in *T. bryosalmonae* infected fish, whereas IgD+IgM- cells are not commonly found in the healthy kidney. In the same study, a repertoire analysis of the three Igs indicated that the fish host response may not involve the clonal selection of a specific B cell subset, but a polyclonal activation of a wide range of B cell subsets (11). These results led the authors to speculate, that as happens in other parasite systems such as *Plasmodium falciparum* (12) *or Trypanosoma cruzi* (13), this polyclonal Ig activation is provoked by the parasite to dilute the specific B cell response. In this setting, ineffective antibody production may be a result of B cell abnormalities induced either directly or indirectly through infection (14). Whether a specific Ig response is mounted during the recovery phase is still unknown.

Despite the huge immunopathological reaction in the rainbow trout posterior kidney during PKD pathogenesis, under certain circumstances infected fish can survive the disease, clear the parasite burden, develop protective immunity and even restore full kidney structure (15, 16). Fish are unique in this ability to completely restore renal structure and functionality from such extensive tissue damage, in contrast to mammals that can only partly restore their nephrons (17). Histopathological reports of PKD survivors have indicated that there is an eventual regression in renal lesions and proliferation and infiltration in the interstitium becomes displaced by fibrotic tissue followed by tissue regeneration during recovery (15). Still, only limited knowledge exists regarding the host immune response which coincides with the clearance of the parasite and progression to recovery as almost all lines of evidence cover advanced clinical or developing PKD. The disease recovery phase is a distinctive course of its own and research into such processes in humans has yielded some fascinating breakthroughs. Illustrating this, Torres et al. (18) used a malaria model to generate disease maps which interpreted the route that individuals take through the disease process as they develop the infection, recover or die. The authors discovered that human malaria patients who are heterozygous for sickle cell hemoglobin occupy a small area of red blood cells by reticulocyte space, proposing that this parameter might be utilized to differentiate resilience at both population and individual levels (18). Similarly, identifying immune mechanisms that correlate with parasite clearance in the PKD model, in contrast to those that coincide with developing or advanced infection, would inform us on which immune mechanisms are clearly anti-parasitic. In this context, analyzing the host response until full parasite clearance would, in combination with earlier studies, unravel how the immune response is regulated during the entire infection progression, from healthy state to recovery, enabling the measurement of an immune response to pinpoint a specific disease stage (18).

In the present study, we utilized the rainbow trout–*T. bryosalmonae* parasite system as a model of disease recovery from a chronic immunopathology. Our aims were to (1) identify when the host transitioned from the plateau of parasite burden to the parasite clearance phase, (2) to temporally examine the histopathological and immune response in the anterior kidney and posterior kidney correlating with this alteration in disease stage. For this, we used a combination of FACS to identify leukocyte populations and subgroups and immune gene expression analysis of transcripts associated with different cellular lineages and functional pathways. We investigated the immune response in both the anterior and posterior kidney.

## Materials and Methods

### Fish Specimens and Experimental Conditions

Fish specimens and parasite exposure procedures are identical as those reported earlier (8). In short, young-of-the-year female rainbow trout, weighing 8–10 g, were sourced from a commercial hatchery in western Switzerland (L'Isle, Switzerland) with no history of PKD. Fish were transported and acclimated for 2 weeks in the aquarium at the Centre for Fish and Wildlife Health, University of Bern, Bern, Switzerland (FIWI). Fish were kept at 15°C, and a control and infection group and a replicate of each were established. Experiments used 130 l flow-through glass tanks supplied with tap-water (~1.l/m), constant aeration and artificial light (12 h light to 12 h dark).

### Parasite Exposure

Freshwater bryozoa (*Fredericella sultana*), the invertebrate host of *T. bryosalmonae*, were collected from Swiss rivers known to be endemic for the parasite, transported to the FIWI and screened for infective parasite sacs by dissection of the bryozoan zooids under a binocular as per previous studies performed in our lab (8, 16, 19). To release the *T. bryosalmonae* spores from the sacs, bryozoan zooid tissue was disrupted by grinding, and the homogenate was kept for 24 h in fresh tap water at room temperature. In addition, DNA was isolated from the homogenate from two 100 ml replicate samples and qPCR was performed to confirm presence of the parasite DNA in the homogenate for infection. Afterwards, in the aquarium tanks, the flow through was stopped, aeration increased, and tank water lowered to around 30%. Equal volumes of the parasite homogenate were then distributed to the infection replicates and after 1.5 h the flow through was restarted. Procedures were performed simultaneously for controls without the addition of parasites. The presence of *T. bryosalmonae* in infected fish was also confirmed by qPCR in the posterior kidney, as described below.

### Fish Sampling and Study Design

Fish sampling procedures were carried out as described in Bailey et al. (8) in which six independent fish were sampled for cellular (FACS), histological and molecular procedures (qPCR/RT-qPCR) per time point from unexposed control fish and *T. bryosalmonae* exposed fish. For cellular procedures, fish were pooled in groups of two due to size of fish at start of study, thus having three biological replicates. Samples were taken over the course of 20 weeks P.E. (post exposure) in which week 0–7 P.E. showed the development of the disease until the plateau of parasite burden (8) while weeks 8–20 P.E. showed the decline of the parasite in the posterior kidney until complete clearance of burden. This latter period is the study period in which the present manuscript has focused. From this time period, samples were taken at weeks 8, 10, 14, and 20 P.E. To allow the reader to understand the change that occurred from week 7 to 8 P.E. relevant data from this earlier time period has been included in our analysis from a previous study from the same source of fish (8). Moreover, as fish at week 20 P.E had for the majority cleared the parasite we did not investigate the immune response in these fish as there was only a limited number of fish to analyze per parameter.

At each sampling, fish were euthanized by immersion into a solution of MS-222 (100 μg/l buffered 3-aminobenzoic acid ethyl ester (MS 222®, Argent Chemical Laboratories). Immediately following blood collection, the entire kidney was removed and sectioned into anterior kidney (AK- the kidney tissue located cranially) and posterior kidney (PK- the kidney tissue located past the narrow site below the neck until the caudal end). Both parts were weighed individually. The PK weight was used to calculate the PK somatic index [PKSI = posterior kidney weights (g)/body weights (g) ×100].

The AK and PK were sampled for all molecular and FACS procedures, while only the PK was used for histology/immunohistochemistry procedures. A DNA sample was taken from the PK of all fish to determine infection intensity. The extracted tissues for DNA/RNA isolation were placed on RNase and DNase free Petri dishes and sliced using scapula and forceps into smaller pieces before being transferred to RNAlater (Qiagen, Basel, Switzerland) on ice for 24 h before storage at −20°C until use. FACS was carried out on the day of sampling. Histology samples were fixed in Histochoice (Amresco, Dietikon, Switzerland) for 3 h at room temperature (RT) and subsequently transferred to graded ethanol (EtOH) (≥99.8–70%). All procedures were carried out according to the Swiss legislation for animal experimentation guidelines and approval for animal experiments was obtained from the cantonal veterinary office (Bern, Switzerland) (Authorization number BE60/14).

### DNA Extraction and qPCR for Determination of Parasite Kinetics

Approximately 25 mg of tissue was used for DNA extraction. DNA was extracted from the PK using the DNeasy blood and tissue kit following the manufacturer's instructions (Qiagen, Basel, Switzerland). qPCR for *T. bryosalmonae* was carried out using a Taq man assay using 250 ng of DNA template per reaction, ran in duplicate as previously reported using *T. bryosalmonae 18s* (Accession number KF805631) using the primers (FWD primer (5′-3′) – GCGAGATTTGTTGCATTTAAAAAG, REV primer (5′-3′) – GCACATGCAGTGTCCAATCG) and probe (CAAATTGTGGAACCGTCCGACTACGA) previously designed by Bettge et al. (20). DNA extracted from control fish was analyzed at every time point which always tested negative for *T. bryosalmonae* in addition to non-target controls (water) within the qPCR that never showed any amplification.

### Histology

Following routine processing and paraffin embedding, PK sections of 3–5 μm thickness were prepared on SuperFrost® Plus positively charged glass slides. After deparaffinization in Histo-Clear (Agar Scientific Ltd, Essex, UK) and rehydration in a graded series EtOH concentrations (≥99.8–50%) the slides were stained with H&E (hematoxylin and eosin) for routine histology. Two slides were evaluated for each fish.

### Isolation and Preparation of Anterior and Posterior Kidney Leukocytes for FACS

Preparation of AK and PK leukocytes was carried out as described earlier (8). In short, the AK and PK was removed from fish and put through a 250 μm mesh filter with Leibovitz's (L-15) medium (Thermofisher, Reinach, Switzerland) containing 10 U/ml heparin. Cell suspensions were then layered onto an isotonic Ficoll gradient (Biochrom AG, Berlin, Germany) (*r* = 1.077 g/ml) and spun at 1,300 RPM for 40 min at 4°C. Cells at the Ficoll/medium interphase were then collected, washed in L-15 medium and resuspended in 1 ml of medium. Red blood cells were then lysed using 9 ml cold distilled water and the cell suspension centrifuged at 1,300 RPM for 1 min at 4°C. Cells were then resuspended in 1 ml L-15 medium supplemented 5% FBS (fetal bovine serum) and kept on ice.

### FACS Analysis of Anterior and Posterior Leukocytes

The monoclonal antibodies (MAbs) MAb1.14 (recognizing membrane-bound trout IgM) and MAbN2 (recognizing the κ-like Ig light chain) were combined in a multiple epitope targeting approach recognizing B cells as earlier described (21, 22). A MAb staining rainbow trout CD8α was also used to study the presence of cytotoxic CD8^+^ T cells as previously described (23). After 30 min of incubation of isolated AK and PK leukocytes with the MAbs diluted in L-15 medium (1:1000) at 4°C, the cells were washed twice with L-15 medium and incubated for 20 min with corresponding secondary Abs. These included, for B cells: R-Phycoerythrin (RPE) conjugated fragments of goat anti-mouse Igg (H+L) and for anti-CD8α RPE conjugated fragments of donkey anti-rat Igg (H+L) (both used at a 1:1000 dilution and obtained from Jackson ImmunoResearch Laboratories, West Grove, PA, USA). Controls incubated with secondary antibody conjugates only were included for each sample. After incubation, cells were washed again two times with L-15 medium. The samples were analyzed on a BD LSR II flow cytometer (San Jose, CA, USA) to a total count of 1 × 10^4^ events. Stained cells were evaluated using the appropriate fluorescence channel for each MAb in comparison to unstained cells and analyzed by Flow Jo version 10 software (TreeStar). Light scattering properties FSC (size) and SSC (granularity) were used to distinguish between lymphocytes and larger granular cells of the myeloid lineage (granulocytes and monocytes/macrophages) as previously described (8, 24).

### Immune Gene Transcript Analysis

Approximately 25 mg of tissue was used for RNA extraction. RNA was extracted from the AK and PK using a Direct-zol™ RNA MiniPrep w/TRI-Reagent® kit following the manufacturer's instructions (Zymo, Freiburg im Breisgau, Germany). Potential traces of genomic DNA contamination were removed with an on column DNAse treatment provided by the kit manufacturers. cDNA was synthesized using The GoScript™ Reverse Transcription System following the manufacturer's instructions (Promega, Madison, Wisconsin, USA). For each sample, 1 μg of DNA-free RNA was used. The total volume of the cDNA syntheses was 20 μl which was diluted 1:10 with Nuclease-Free water (Promega, Madison, Wisconsin, USA) and stored at −20°C until analysis.

To evaluate gene transcription levels, RT-qPCR was performed with a LightCycler® 96 System instrument (Roche) using SYBR Green PCR core Reagents (Promega) and specific primers previously optimized (Table S1). All samples were measured in duplicate. The total reaction volume was 20 μl, containing 10 μl GoTaq® qPCR Master Mix, 1 μl of 10 μM primer stocks (final concentration 500 nM), 3 μl Nuclease-Free water and 5 ul of the diluted cDNA synthesis mix. RT-qPCR was performed using the following settings: 5 min 95°C, followed by 40 cycles of amplification. Each cycle consisted of 3 s of denaturation at 95°C, annealing and elongation at 60°C for 30 s. The PCR was always terminated with a melting curve analysis starting with a denaturation step of 95°C followed by the start ramping temperature of 60°C for 30 s. The data were analyzed with the Roche light cycler 96 Application Software Version 1.1. Data was evaluated according to the 2–^ΔΔ*Ct*^ method (25) using the rainbow trout reference gene elongation factor 1α (*ef-1*α) (see Figure S1 for reference gene validation). No template negative controls and *minus* reverse transcriptase controls were included in all assays.

### Statistics

The differences between the control and infection group at each time point in the AK and PK were tested for significant differences using the two-tailed Student *t*-test. The differences between infected fish sampled at the different time points were tested for, using a one-way ANOVA and significant differences revealed with the Dunnett's *post-hoc* test. Data failing normality tests and displaying heterogeneity of variance were tested statistically applying the non-parametric Kruskal–Wallis ANOVA on ranks, and Dunn's non-parametric multiple comparison tests to reveal differences. Gene expression data was tested statistically at the ΔCt stage before the log transformation. Correlations between parasite intensity, PKSI, and immune gene expression (using log transformed data) were assessed by calculating the Pearson correlation coefficient (*r*) in the *T. bryosalmonae* infected fish. Data were statistically evaluated with SigmaPlot 12.0 (Systat Software, San Jose, CA) and graphically presented with GraphPad Prism 5 (GraphPad Software, Inc. San Diego, CA). Significance was set at *p* ≤ 0.05 and *p*-values below < 0.005 and < 0.001 were indicated as such in the data presentation.

## Results

### Parasite Intensity Declined 1 Week After Maximum Burden Is Reached

**Figure 1:**
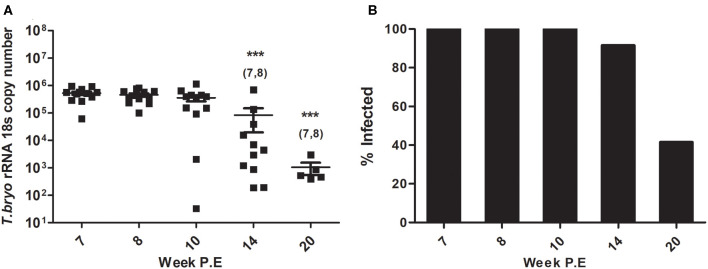
**(A)** Declining parasite intensity (Arithmetic Mean ± SE) and **(B)** infection prevalence (mean percentage of infected fish sampled per time point) during recovery in *Tetracapsuloides bryosalmonae* infected rainbow trout, starting at peak of parasite burden [week 7 P.E (post-exposure)]. Parasite intensity was expressed as 18S rRNA gene copies of *Tetracapsuloides bryosalmonae* in rainbow trout posterior kidney sampled at week P.E. Numbers above time points in **(A)** indicate at which week P.E the time point was significantly decreased in comparison to (week 7 P.E) (ANOVA). Number of asterisks indicates level of significance (*p < 0.05, **p < 0.005, and ***p < 0.001) relative to infected fish sampled at the specific time point. *N* = 12 per time point unless fish were uninfected in which there was no burden i.e., week 14 P.E, *N* = 11, or week 20 P.E, *N* = 5.

At week 8 P.E, *T. bryosalmonae* intensity in the PK began declining in comparison to week 7 P.E. which was the plateau of parasite intensity when the parasite had appeared to reach its maximum burden within the fish host (Figure 1A). In fact, at all weeks P.E. tested after week 7 P.E there was a decline in parasite intensity in contrast to this week P.E. Although we did not include week 7 P.E in the immune gene analysis reported here, we included the parasite intensity data and also cellular data in this manuscript for the reader to understand our experimental design focusing our study from the start of parasite clearing (week 8 P.E). At week 14 and 20 P.E there were significant decreases in parasite intensity relative to week 7 and week 8 P.E.

Presence of the parasite was no longer detectable in some fish and subsequent reduction in infection prevalence occurred (mean percent of infected fish sampled per time point). Initially all rainbow trout sampled at week 8 P.E, and week 10 P.E tested positive for *T. bryosalmonae* infection (12/12 fish sampled) as per earlier time points (Figure 1B). Eventually, at week 14 P.E the first fish tested negative for presence of the parasite, while at week 20 P.E infection prevalence had declined to <50% (5/12 fish sampled). It must also be noted that this prevalence at late time points might be even lower, as it could be possible fish with low parasite loads have in fact no viable parasites and that our qPCR method is in fact detecting dead or decayed *T. bryosalmonae*. To date, however, it is not possible for us to distinguish between live and dead parasites.

### Onset of Fibrosis but Not a Decrease in Renal Inflammation Indicated Start of Posterior Kidney Regeneration Phase

**Figure 2:**
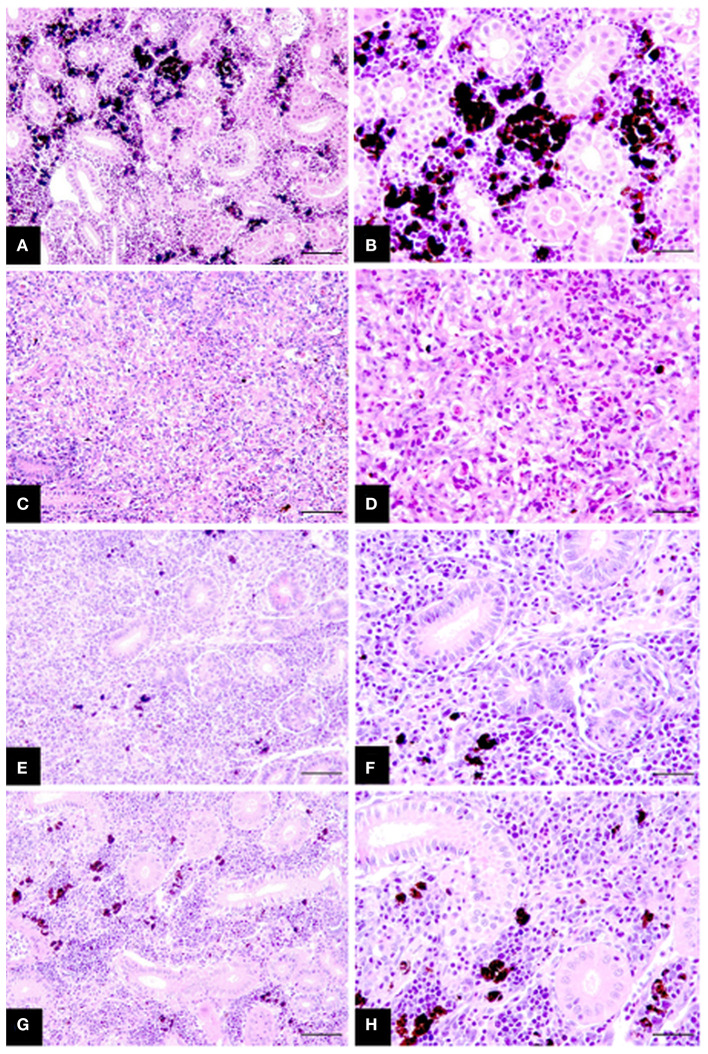
Chronicling of rainbow trout recovery from *T. bryosalmonae* infection in the posterior kidney **(A–H)**. **(A,B)** Posterior kidney section of healthy uninfected fish showing intact tubules and abundant melanomacrophages. **(C,D)** Infected fish at week 8 P.E with strong tissue proliferation, almost complete deterioration of tubules, deposits of fibrosis tissue and scattered parasites. **(E,F)** Infected fish sampled at week 10 P.E exhibiting increased signs of tissue recovery including tubuloneogenesis and infrequent patches of melanomacrophage. However, there is still some presence of parasites but this is greatly reduced compared to **(C,D)**. **(G,H)** Infected fish sampled at week 14 P.E showing advanced regeneration and resolution of tubules and abundant melanomacrophages in tissue but not yet completely similar to healthy uninfected fish in **(A,B)**. H&E stain; bars 50 μm **(A,C,E,G)** and 25 μm **(B,D,F,H)**.

Collectively histological changes in infected fish showed a steady regression in parasite presence and tissue proliferation becoming displaced with fibrosis tissue, tubuloneogenesis and re-emergence of melanomacrophage centres. However, these changes were often patchy in form occurring in gradients throughout the sections and not systemic, increasing slowly over time starting at week 8 P.E (Figure 2). In this setting, fish at week 8 and 10 P.E still had histology consistent with advanced PKD and large areas were observed with a complete deterioration of renal tubules. While even at week 14 P.E on occasion the presence of parasites was visible although sometimes these were degenerative in form characterized by hypereosinophilia and fragmentation of parasite cells and nuclei.

**Figure 3:**
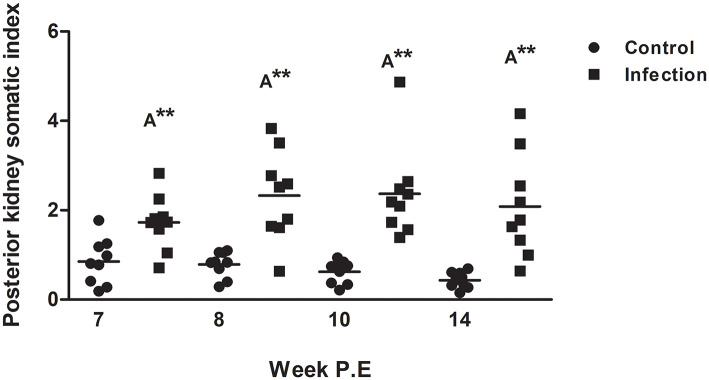
Posterior kidney somatic index of *Tetracapsuloides bryosalmonae* infected rainbow trout and unexposed control fish (Scatter dot plots with Median ± SE at week post exposure) (P.E). Letter above data plots indicate significant increase to controls (A), while, the number of asterisks indicates level of significance (*p < 0.05, **p < 0.005, and ***p < 0.001). *N* = 9 per time point, per control and infection treatments.

The PKSI of infected fish was significantly greater at every time point in comparison to the control although there were no significant differences when comparing infected fish at different time points (Figure 3). Surprisingly at week 8, 10, and 14 P.E the PKSI even increased relative to week 7 P.E as the parasite burden was declining, indicating an increase in inflammation before beginning to decrease slightly at week 14 after week 10 P.E. The increase in PKSI was possibly a by-product of the initial alteration in tissue composition caused by the onset of fibrosis as observed from the histology.

### Gradual Decrease of Lymphocytes and Increase of Myeloid Cells Occurred During Declining Parasite Intensity

**Figure 4:**
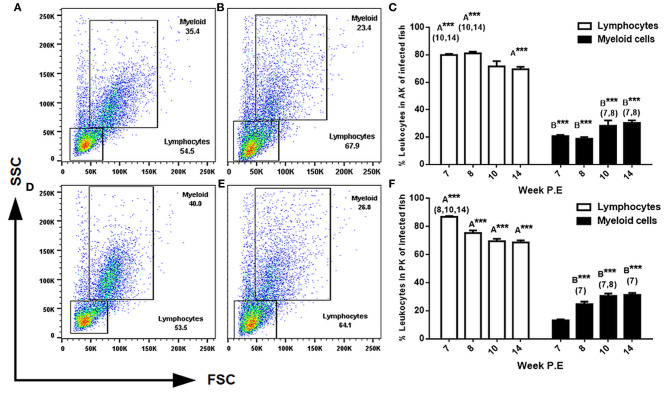
FACS analysis of AK (anterior kidney) and PK (posterior kidney) leukocytes in *T. bryosalmonae* infected rainbow trout. Representative FSC (size) and SSC (granularity) dot plots showing the gating strategy in the AK of uninfected **(A)** or infected **(B)** fish and in the PK of uninfected **(D)** and infected **(E)** fish at week 14 P.E. Bar charts showing percent of leukocytes in infected fish in the AK **(C)** and PK **(F)** of 10^4^ gated events (Arithmetic Mean ± SE). Letters above data plots indicate significant increase to controls **(A)** or significant decrease to the controls **(B)** (*t*-test). The number of asterisks indicates level of significance (*p < 0.05, **p < 0.005, and ***p < 0.001). Numbers above time points in which values were significantly increased in comparison to infected fish from other time points (ANOVA). *N* = 3 per time point, per control and infection treatments.

The two most reported cellular features of PKD pathogenesis are a decrease in myeloid cells and the *in-situ* proliferation of lymphocytes in the AK and PK (7, 8). Thus, we expected a shift in these cellular dynamics in the present study during recovery. To evaluate this, we used light scattering properties FSC (size) and SSC (granularity) to distinguish between lymphocytes and larger granular cells of the myeloid origin in the AK and PK. In general, while there was a pattern in infected fish in both the AK and PK to have significantly more lymphocytes and less myeloid cells than the respective control, a subsequent decrease in lymphocytes and an increase in myeloid cells in comparison to infected fish sampled at earlier time points began to transpire as parasite intensity declined indicating the beginning of a shift in cellular kinetics in infected fish (Figure 4). For example, in the AK at weeks 7, 8, and 14 P.E there was a significantly greater number of lymphocytes relative to the control. But at weeks 10 and 14 P.E there was a significant decrease in lymphocytes in comparison to infected fish at weeks 7 and 8 P.E. In the PK there were also a significantly greater number of lymphocytes at each time point (week 7, 8, 10, and 14 P.E) in comparison to the control. However, between the infected fish there was a significant decrease in lymphocytes over time (week 8, 10, and 14 P.E vs. week 7 P.E).

Regarding the assessment of myeloid cells in the AK and PK at every time point there were significantly less myeloid cells found in these tissues in infected fish (at weeks 7, 8, 10, and 14 P.E) relative to the controls. However, again, the population of myeloid cells in both the AK and PK significantly increased over time in the infection groups as at week 10 and 14 P.E there were significant increases relative to week 7 and 8 P.E.

### Diminished Presence of B Cells in the AK and PK Occurs During Host Recovery

**Figure 5:**
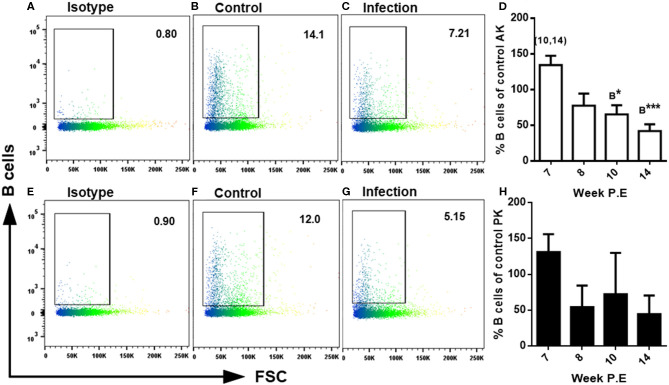
FACS analysis of rainbow trout AK (anterior kidney) and PK (posterior kidney) leukocytes using monoclonal antibodies staining B cells in *Tetracapsuloides bryosalmonae* infected rainbow trout. Representative dot plots of isotypes in the AK **(A)** and PK **(E)** and the levels of B cells measured in the AK of uninfected fish **(B)** and in the AK of infected fish **(C)** and in the PK of uninfected fish **(F)** and in the PK of infected fish **(G)** all at week 14 P.E. Bar charts showing percent of B cells as percentage of control in infected fish at week post exposure (P.E) (Arithmetic Mean ± SE) in the AK **(D)** and PK **(H)**. Letters above data plots indicate significant increase to controls **(A)** or significant decrease to the controls **(B)** (*t*-test). The number of asterisks indicates level of significance (*P < 0.05, **P < 0.005, and ***P < 0.001). Numbers above time points indicate which time point was significantly increased in comparison to in infected fish (ANOVA). *N* = 3 per time point, per control and infection treatments.

During developing PKD pathogenesis strong increase of all three fish Igs (IgM, IgD, and IgT) at the protein level has been documented (11). For a characterization of the cellular composition in the present study lymphocyte subsets were identified using MAbs staining B cells and CD8α^+^ T cells via FACS. Regarding the measurement of B cells at week 7 P.E in both the AK and PK there was a greater amount of B cells measured in infected fish, though at week 8 P.E this began to decrease and less B cells were found in infected fish in the AK and PK in comparison to the control with significantly less stained cells found in the AK at week 10 and 14 P.E in comparison to the controls. In addition, there was a significant decrease in B cells in the AK of infected fish at week 10 and 14 P.E in comparison to week 7 P.E (Figure 5). In addition, in the PK at week 8, 10, and 14 P.E there was a massive decline in the B cells levels of infected fish in comparison to uninfected fish and to infected fish sampled at week 7 P.E. While concerning CD8α+ T cells detected using FACS there was a significantly lower amount of these cells detected in the AK at week 10 P.E in comparison to the control but there were no significant differences between the infected fish in either the AK or PK at any time points (data not shown).

### Regulation of Immune Gene Transcripts During Parasite Clearance and Host Recovery

#### Innate Immune Markers

**Figure 6:**
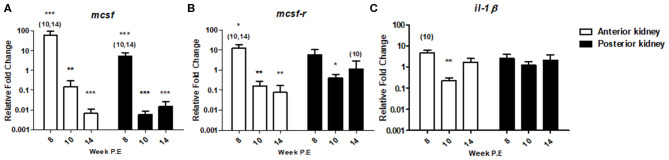
Regulation of innate immune gene transcripts. Relative fold change of **(A)** *mcsf* ; **(B)** *mcsf-r*; and **(C)** *il-1*β; measured in the AK and PK of *T. bryosalmonae* infected fish at different weeks post exposure (P.E) (Arithmetic Mean ± SE). Relative fold change was normalized to rainbow trout reference gene *ef-1*α and subsequently expressed as fold change relative to expression levels of control fish. Asterisks indicate differences relative to the control (*t*-test). Number of asterisks indicates level of significance (*p < 0.05, **p < 0.005, and ***p < 0.001). Numbers above time points indicate which time point was significantly increased in comparison to other infected time points (ANOVA). *N* = 6 per time point, per control and infection treatments.

In *T. bryosalmonae* infected fish, gene expression studies have demonstrated the putative lack or transient response of pro-inflammatory cytokines and inhibition of markers of the myeloid response (8–10). To determine how markers encoding for such mechanisms might change during recovery we measured *mcsf* a mediator involved in regulating the numbers and functions of cells from the myeloid lineage and which may contribute to myeloid heterogeneity as well as its receptor *mcsf*-*r* and the traditional pro-inflammatory cytokine *il-1*β (Figure 6). At week 8 P.E, —*mcsf* [fold change (fc): 61.42] and *mcsf-r* (fc: 12.40) were significantly upregulated in the AK relative to the control as well in comparison to infected fish at week 10 and 14 P.E. In addition, *mcsf* was significantly upregulated in the PK at week 8 P.E in comparison to the control. However, at week 10 and 14 P.E there was a drastic shift in the expression profiles of these transcripts in the AK as *mcsf* and *mcsf*-*r* were (fc: 0.14 and 0.16, respectively) significantly downregulated relative to the control. Furthermore, *mcsf* was strongly downregulated at these time points in the PK as was *mcsf-r* at week 10 P.E relative to their respective controls. Such intense fluctuations did not occur in the expression level of *il-1*β as there was only a small significant decrease in the mRNA levels of this gene at 10 P.E relative to the control in the AK, whereas there were no significant differences concerning other time points in the AK or in the regulation of this gene in PK.

#### B Cell Transcripts

**Figure 7:**
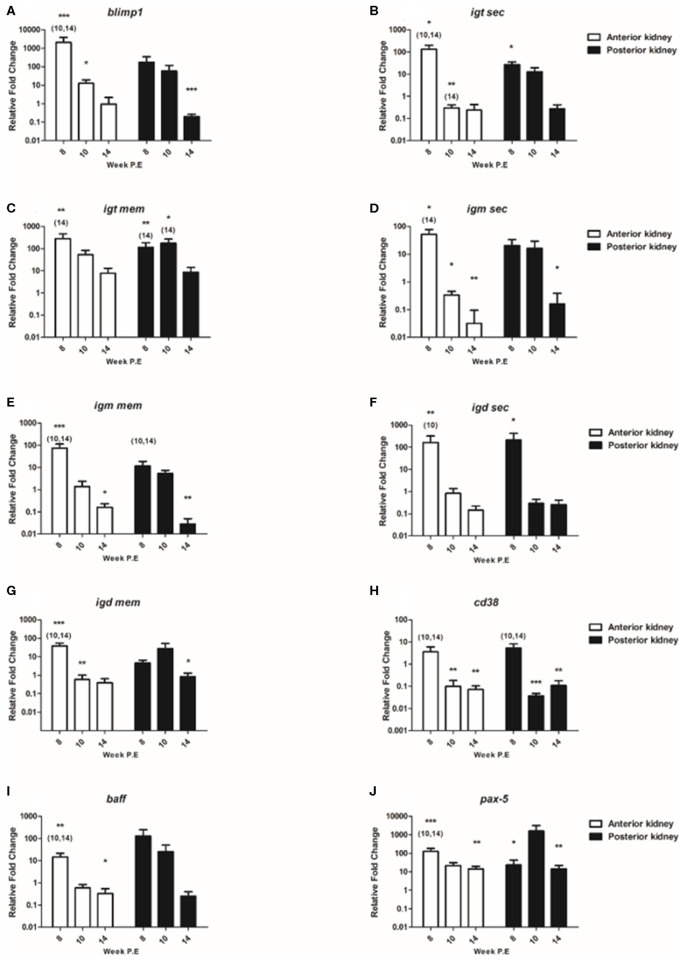
Regulation of B cell transcripts. Relative fold change of **(A)** *blimp-1*; **(B)** *igt sec*; **(C)** *igt mem*; **(D)** *igm sec*; **(E)** *igm mem*; **(F)** *igd sec*; **(G)** *igd mem*; **(H)** *cd38*; **(I)** *baff;* and **(J)** *pax-5* measured in the anterior and posterior kidney of *T. bryosalmonae* infected fish at week post exposure (P.E) (Arithmetic Mean ± SE). Relative fold change was normalized to rainbow trout reference gene *ef-1*α and subsequently expressed as fold change relative to expression levels of control fish. Asterisk indicates differences relative to the control (*t*-test). Number of asterisks indicates level of significant (*p < 0.05, **p < 0.005, and ***p < 0.001). Numbers above time points indicate which time point was significantly increased in comparison to (ANOVA). *N* = 6 per time point, per control and infection treatments.

In the present study we composed a panel of B cell markers consisting of the secreted and membrane forms of the three fish Igs (*igm sec, igd sec, igt sec, igm mem, igd mem, igt mem*) and of *blimp1, cd38, baff*, and *pax-5* (Figure 7). In general, the mRNA levels of these B cell markers decreased over time as parasite intensity reduced in infective fish. For instance, at week 8 P.E regarding *blimp1* in the AK of infected fish the mRNA levels of this gene were strongly significantly elevated (fc: 2078.4) relative to the control and in infected fish sampled at week 10 and 14 P.E. While *blimp1* was still significantly upregulated in the AK at week 10 P.E, expression levels were severely decreased (fc: 12.6) before becoming downregulated at week 14 P.E. The same expression pattern was followed in the PK for *blimp1*, declining temporally from week 8 P.E (fc: 177.4) to week 14 P.E (fc: 0.2) in which the gene was significantly downregulated in comparison to the control.

Concerning the genes measured encoding for the different fish Igs; *igt sec* was significantly upregulated in the AK and PK at week 8 P.E relative to the control. Nevertheless, at week 10 and 14 P.E in the AK the mRNA levels of *igt sec* decreased significantly in contrast to the control and to infected fish sampled at 8 weeks P.E. Regarding *igt mem* the expression of this transcript was significantly stronger expressed at week 8 P.E in both the AK and PK relative to the control fish and infected fish sampled at week 14 P.E in both organs. In addition, in the PK this transcript was significantly upregulated at week 10 P.E in comparison to the control and *T. bryosalmonae* infected fish sampled at week 14 P.E. It must also be stated here that despite mRNA levels decreasing somewhat over time this was the only Ig transcript membrane bound or secreted that remained moderately upregulated in both tissues even at week 14 P.E. Exemplifying this, *igm sec* was significantly upregulated in AK at week 8 P.E but significantly downregulated at week 10 and 14 P.E and also at week 14 P.E in the PK relative to the control. In a similar fashion, *igm mem* was significantly elevated in AK at week 8 P.E but downregulated at week 14 P.E in the AK and PK relative to the control. Furthermore, the expression levels of *igm mem* were significantly downregulated in the AK and PK in infected fish at week 10 and 14 P.E in comparison to infected fish at week 8 P.E. Pertaining to *igd sec*, the expression level of this gene was significantly upregulated in the AK at week 8 P.E and strongly significantly elevated at week 8 P.E in the PK before becoming downregulated in both the AK and PK at week 10 and 14 P.E. While *igd mem* transcripts were significantly elevated in the AK at week 8 P.E, in contrast to the respective control and to infected fish sampled at week 10 and 14 P.E.

Additionally, included in the panel of B cell markers was *cd38*, which in mammals is suggested to be consistently expressed on terminally differentiated plasma cells (26). At week 8 P.E there were significantly greater mRNA levels of this gene measured in the AK and PK of infected fish in comparison to infected fish sampled at week 10 and 14 P.E. Eventually, the mRNA levels of *cd38* significantly declined in the AK and PK of infected fish at week 10 and 14 P.E with the transcript being downregulated in comparison to the control. Regarding the expression levels of *baff* the B cell activation factor, in the AK and PK the expression levels of this gene also declined drastically temporally. In this perspective, this gene was significantly upregulated in the AK at week 8 P.E in comparison to uninfected control fish and infected fish sampled at week 10 and 14 P.E. There were no significant differences concerning the expression of this gene in the PK.

Additionally, we measured *pax-5*, which in humans encodes the B cell lineage specific activator protein (Bsap) that is suggested to be expressed at early, but not late stages of B cell differentiation (27). The expression of this gene in infected fish was significantly stronger expressed in the AK at week 8 P.E (fc: 128.4) in relation to the uninfected control fish and to infected fish sampled at week 10 and 14 P.E. As per *igt mem, pax-5* still remained upregulated at week 14 P.E in both organs and it was one of the only genes (along with *il-10*) to be significantly upregulated at week 14 P.E in both the AK and PK in contrast to the controls.

#### T Cell Transcripts

**Figure 8:**
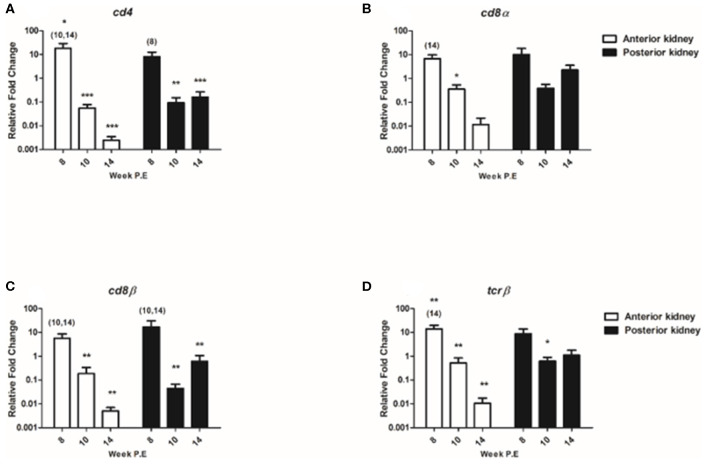
Regulation of T cell transcripts. Relative fold change of **(A)** *cd4*; **(B)** *cd8*α; **(C)** *cd8*β*;* and **(D)** *tcr*β measured in the anterior and posterior kidney of *Tetracapsuloides bryosalmonae* infected fish at week post exposure (P.E) (Arithmetic Mean ± SE). Relative fold change was normalized to rainbow trout reference gene *ef-1*α and subsequently expressed as fold change relative to expression levels of control fish. Asterisk indicates differences relative to the control (*t*-test). Number of asterisks indicates level of significant (*p < 0.05, **p < 0.005, and ***p < 0.001). Numbers above time points indicate which time point was significantly increased in comparison to (ANOVA). *N* = 6 per time point, per control and infection treatments.

The regulation of T cell signature molecules (*cd4, cd8*α, *cd8*β, and *tcr*β) were evaluated (Figure 8). Preceding PKD pathogenesis transcriptional studies have thus far only indicated a moderate involvement of these generalist T cell genes in contrast to B cell transcripts (8, 10). In the present study, while *cd4* was significantly upregulated in the AK at week 8 P.E the mRNA levels of *cd4* eventually decreased at week 10 and 14 P.E as the transcript became significantly downregulated relative to the control in both the AK and PK. While *cd8*α was only moderately expressed or downregulated in both organs throughout the study with expression levels being significantly downregulated at week 10 P.E in comparison to the control. The expression signatures of *cd8*β and *tcr*β followed a similar pattern with *cd8*β expression being significantly greater in the AK and PK of infected fish at week 8 vs. 10 and 14 P.E and *tcr*β expression levels being upregulated in the AK at week 8 in comparison to the control and infected fish at week 14 P.E prior to a significant decline in both of these transcripts. In this perspective, *cd8*β became significantly downregulated in the AK and PK at week 10 and 14 P.E vs. control and *tcr*β in the AK at the same time and at week 10 P.E in the PK vs. the control.

#### Immunoregulatory Checkpoint Molecules and Master Transcription Factors

**Figure 9:**
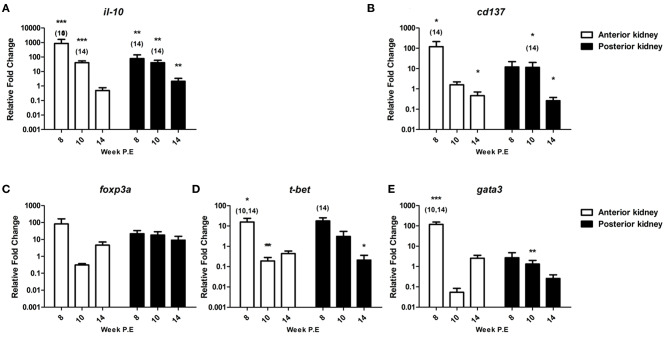
Regulation of immunoregulatory checkpoint molecules and master transcription factors. Relative fold change of **(A)** *il-10*; **(B)** *cd137*; **(C)** *foxp3a*; **(D)** *t-bet;* and **(E)** *gata3* measured in the anterior and posterior kidney of *T. bryosalmonae* infected fish at week post exposure (P.E) (Arithmetic Mean ± SE). Relative fold change was normalized to rainbow trout reference gene *ef-1*α and subsequently expressed as fold change relative to expression levels of control fish. Asterisk indicates differences relative to the control (*t*-Test). Number of asterisks indicates level of significant (*p < 0.05, **p < 0.005, and ***p < 0.001). Numbers above time points indicate which time point was significantly increased in comparison to (ANOVA). *N* = 6 per time point, per control and infection treatments.

*il-10* is an immunoregulatory cytokine that has been shown to be hyper upregulated in earlier transcriptional studies during PKD pathogenesis (8, 10, 28). In the present study, the mRNA levels of *il-10* were also very strongly expressed. As a case in point, in the AK at week 8 P.E (fc: 865.7) and PK (fc: 77.4) at week 8 P.E (Figure 9). The mRNA levels measured at this time point in the AK and PK as well as at week 10 P.E were significantly higher than the controls and to infected fish sampled at week 14 P.E. Likewise, the gene was significantly upregulated at week 14 P.E in the PK relative to the control fish sampled at this time point.

Furthermore, we evaluated the expression of *cd137* a reported co-stimulatory immune checkpoint molecule. Accordingly, CD137 modulates not only the activation state of T cells but activation, proliferation, survival, apoptosis, and differentiation of many immune and non-immune cells and the course of immune response (29) (Figure 9). The transcription levels of this gene were significantly upregulated in AK at week 8 P.E relative to the controls and in comparison to infected fish sampled at week 14 P.E, while the gene was moderately upregulated in PK and at week 8 and 10 P.E, but this was not significant. The mRNA levels of *cd137* decreased at week 14 P.E. in both organs becoming significantly downregulated in the AK and PK in comparison to the control.

We also assessed *foxp3a* transcription, this gene functions as a master transcription factor of the regulatory pathway in the development and function of regulatory T cells which are suggested to generally turn the immune response down (30). *foxp3a* was strongly expressed at week 8 P.E in the AK before becoming downregulated (week 10 P.E) and moderately expressed (week 14 P.E) (Figure 9). While in PK, in contrast to most of the mRNA data generated in this study the levels of *foxp3a* did not strongly fluctuate and were somewhat comparable (upregulated at week 8 fc: 21.7, week 10 fc: 18.1 and week 14 fc: 9.0 P.E, respectively) at every time point.

During PKD pathogenesis an imbalanced Th-1 and Th-2 like profile has been described (8, 10). To see if recovery correlated with a skewing to either of these phenotypes, we examined the master transcription factors of each of these functional pathways *t-bet* (Th-1 like) and *gata3* (Th-2 like) (Figure 9). *t-bet* was significantly elevated at week 8 P.E in the AK in comparison to the controls and relative to infected fish sampled at week 10 and 14 P.E, before becoming significantly downregulated at week 10 P.E in the same organ in comparison to the controls. While in the PK this molecule was significantly elevated at week 8 P.E in comparison to infected fish sampled at week 14 P.E and in the same organ significantly downregulated relative to the controls at week 10 P.E. Regarding *gata3*, the Th-2-like master transcription factor was significantly upregulated at week 8 P.E in AK relative to the controls and infected fish at week 10 and 14 P.E. While in PK at week 10 P.E, *gata3* was significantly downregulated.

#### Regulation of Immune Gene Transcripts Correlating With Parasite Intensity and Pathological Alterations

One transcript in the AK (*pax-5*) and nine (*mcsf* , *mcsf-r, igd sec, cd38, cd4, cd8*α, *cd8*β, *tcr*β*, and foxp3a*) in the PK had a significant positive correlation with parasite intensity, indicating that the expression of these genes significantly decreased with the decline of the parasite burden (Table 1). All the T cell signature molecules we measured correlated in the PK positively with parasite intensity. However, no genes investigated in this study were positively correlated with PKSI. An explanation for this is that the parasite intensity significantly decreased when comparing burdens at different post exposure time points but the PKSI only had significant differences relative to the control and not between infected fish sampled at different time points.

**Table 1:** Summary of significant correlations between parasite intensity and immune gene expression in the anterior and posterior kidney of *Tetracapsuloides bryosalmonae* infected rainbow trout, assessed by calculating the Pearson correlation coefficient (*r*).

**Gene**	**Pearson correlation (*r*^**2**^)**	**Tissue**
*pax-5*	0.489^*^	Anterior kidney
*mcsf*	0.559^*^	Posterior kidney
*mcsf-r*	0.557^*^	Posterior kidney
*igd sec*	0.558^*^	Posterior kidney
*cd38*	0.600^*^	Posterior kidney
*cd4*	0.611^**^	Posterior kidney
*cd8α*	0.545^*^	Posterior kidney
*cd8β*	0.591^*^	Posterior kidney
*tcrβ*	0.622^**^	Posterior kidney
*foxp3a*	0.504^*^	Posterior kidney

Number of asterisks indicates level of significant (

* *P < 0.05*,

** P < 0.005, and ***P < 0.001). N = 17 per correlation assessment.

## Discussion

Recovery is a set of three overlapping processes: pathogen clearance, inflammation resolution and repair of damaged tissue. Here we assessed the immune response in fish beginning to recover from PKD pathogenesis, when pathogen clearance was commencing. To this end, fish were exposed to *T. bryosalmonae* and parasite dynamics in the host evaluated by qPCR with the parasite burden plateauing at week 7 post exposure (P.E) and starting to slightly decline at week 8 P.E and being completely cleared in almost all of the population week 20 P.E. This time period (week 8–20 P.E) was identified as the recovery/parasite clearance phase and the immune response in fish at week 8–14 P.E was evaluated both temporally and correlating with decreasing parasite intensity and alterations in pathology. We investigated the immune response in both the anterior kidney (AK) and posterior kidney (PK). The AK has no renal function and lacks nephrons and is the primary site for lymphohaematopoiesis where B cells develop and where most proliferating B cell precursors are located (19). The PK possesses both renal and immune tissues, hosting substantial populations of partially activated B cells and plasmablasts (19). Importantly, the immune response has been characterized during PKD pathogenesis in these two organs in our previous work (7) allowing a comparison with earlier stages of PKD pathogenesis necessary to elucidate differences between these disease phases. While the pathology associated with recovery had been described in rainbow trout infected with *T. bryosalmonae* before (15), the immune response, both temporally and correlating with parasite clearance had not yet been fully elucidated in rainbow trout. Our results demonstrated that recovery from PKD in young-of-the-year rainbow trout is preceded by an initial intense immune response in both the anterior and posterior kidney at least on the transcript level, a small decrease in parasite burden, but an increased degree of kidney inflammation involved in tissue regeneration.

Given the lymphocytic hyperplasia and hyperimmunoglobulinemia reported during PKD pathogenesis (7), we expected that concerning the immune response (a) a shift in the cellular dynamics (a marked decrease in lymphocytes and an increase in myeloid cells) would occur and that (b) a reduction in the expression of markers for activated plasma cells would transpire. Pertaining to (a) there was a significant decrease in lymphocytes and a significant increase in myeloid cells occurring over time, thus providing evidence of a shift in cellular dynamics. Yet, this occurred much slower than expected and the ratio of these cells remained significantly increased relative to uninfected fish indicating that it will take longer than the study period until a homeostatic return is complete. Pertaining to (b) at week 14 P.E there was downregulation of several transcripts used as markers for plasma cells (*blimp1, igm sec, igt sec, igd sec*, and *cd38*) in both the anterior and posterior kidney and a decrease in in levels of B cells measured using FACS and on the other hand *pax-5* a gene expressed on all B cell stages apart from plasma cells was significantly upregulated (31). This expression profile indicated a change in B cell processes during recovery in terms of an arrest in the strong antibody response transitioning to a profile associated with resting B cells.

These alterations observed in host B cell processes could be speculated to participate in either the clearance of the parasite, the development of protective immunity or through restoring B cell homeostasis via reestablishing lineage identity after aberrant plasma cell production. However, as rainbow trout are a dead end host, given that parasite intensity declined shortly after maximum burden was reached [≈week 7 P.E. as per (8, 20)] at a comparable time in which the parasite has been reported to start shedding from its natural host [the brown trout at day 45 P.E (32)] it could be questioned whether this is a successful host immune response or if the parasite declines or deteriorates due to its inability to complete its life cycle in this host. Furthermore, concerning the B cell response, we saw both a decrease in the plasma cell markers and an upregulation of naïve/resting B cell markers on the transcript level. Illustrating this, there was upregulation of *pax-5* and *igt mem* at week 14 P.E in the AK and PK in contrast to the downregulation of *blimp1*, secreted Igs and also of *cd38* all of which are expressed on plasma cells whereas membrane bound Igs and *pax-5* are expressed on naïve/resting B cells (27, 31). In an earlier PKD study, *pax-5* was only moderately expressed in comparison to *blimp1* during advanced PKD pathogenesis (8). While fish re-exposed to the parasite exhibited a strong early protective immune response that consisted of vigorous *igm sec* expression but downregulation of *pax-5* (16). These fish also had lower pathogen burden and greatly reduced pathology in contrast to fish in the same study exposed to the parasite for the first time (16). Taking the above into account and given the *pax-5* expression profile observed here it might be possible to say that this transcript plays a role in restoring B cell homeostasis through reestablishing lineage identity after aberrant plasma cell production. Thus, the decrease in expression patterns associated with plasma cells and decline of B cells when the parasite burden was decreasing could function in two dimensions: (1) to provide space for new naïve B cells, and/or (2) in terms of immunological investment be unnecessary after removal or deterioration of the parasite.

The B cell response that initiated recovery here extends the findings of Chilmonczyk et al. (7), Abos et al. (11), and Granja et al. (33) which all provided some evidence of a decrease of B cell mechanisms during advanced PKD pathogenesis. For example, Chilmonczyk et al. (7) showed that at week 16–20 post -*T. bryosalmonae* infection lymphocyte populations in the rainbow trout kidney had started to slowly decrease, whereas myeloid cells had begun to slowly increase. Moreover, that these proliferating lymphocytes in the kidney did not consist of increased levels of IgM^+^ B cells when compared to the control (7). Similar outcomes were observed in the present study. Abos et al. (11) reported that the presence of IgM, IgD, and IgT+ B cells were greater in rainbow trout with grade 1–2 clinical swelling than in kidneys in which the disease pathology had progressed to grade 3–4 swelling. While, Granja et al. (33) reported a decreasing trend in *baff* transcripts correlating with levels 3–4 of kidney swelling grade. Likewise, in the present study there was a significant decrease in *baff* transcripts over time. Thus, these earlier studies hinted at a change or reduction in the intensity of the B cell response during clinical PKD although not to the extent or manner as in the present study. Additionally, in the study by Abos et al. (11) the authors reported as that both IgT^+^ cells observed using immunohistochemistry and total *igt* transcripts were dominant relative to measurements of IgM and IgD. Our findings corroborated this somewhat as even at week 14 P.E *igt mem* was still significantly upregulated in both the AK and PK but neither *igd* or *igm* membrane nor secretory transcripts were. In the same study, the authors also provided evidence of the involvement of IgD, but this was most prominently observed at the functional level in the PK and not strong transcriptionally (11). Here we found both *igd mem* and *igd sec* transcripts significantly elevated in the AK at week 8 P.E relative to the controls but not in PK. In an earlier report attempting to characterize plasma cell populations Ramirez-Gomez et al. (34) found that secretory IgD plasma cells are primarily located in the rainbow trout AK in comparison to the PK and, that *igd mem* and *igd sec* transcripts were more abundant in the AK than the PK. Which may explain the differences we found here, although Ramirez-Gomez et al. (34) only investigated fish that were not exposed to any pathogens. In this context, it would have been of further interest to explore the presence of IgD+ B cells using FACS in the AK during PKD pathogenesis, especially given the broader immune role this phenotype has been shown to play in recent rainbow trout studies (35).

Although a body of work has unraveled some of the B cell mechanisms involved in PKD pathogenesis (8, 10, 11, 16, 33), still less is known about the role of T cell subsets. We did not see an increase of any T cell markers in terms of CD8α+ T cells in the FACS analysis or in the mRNA levels of *cd4, cd8*α, *cd8*β, and *tcr*β or of Th-1 or Th-2-like master regulators (*t-bet, gata3*) in the AK and PK during recovery. Though, the decrease of *cd4, cd8*α, *cd8*β, and *tcr*β transcript levels did significantly correlate with the decrease of parasite intensity in the PK which hints at an earlier role for T cells. A previous study investigating PKD pathogenesis solely on the transcript level in rainbow trout also reported a correlation between *cd4, cd8*α, and *cd8*β elevation and parasite burdens (10). Still further research during PKD pathogenesis is needed concerning the role of T cells particularly at the functional level that goes beyond what we reported on here and in previous studies.

The two most notable cellular changes during the development of PKD pathogenesis is a decrease in myeloid cells and an increase in lymphocytes (7, 8). Here we showed a temporal trend of decreasing lymphocytes and increasing myeloid cells within the AK and PK during the late stage of the disease although we expected this process would occur much faster. Histologically we observed an increased presence of melanomacrophage centres which is in agreement with an increase in macrophages that has already been reported in fish recovering from PKD (15). Additionally, at the transcription level we observed strong upregulation of *mcsf* but only at week 8 P.E in the AK. It could be presumed that as the disease recovery continues an upregulation of macrophage markers or M2 phenotypes could feature in tissue resolution. For instance, in a late stage PKD investigation (taking place solely at day 130 P.E = 19.5 weeks P.E) using an RNA-seq approach, *slc11a1* (formerly known as *nramp*α) and *mcsf* were both significantly upregulated (relative to the control) in the PK of fish exposed to *T. bryosalmonae* and an endocrine disrupting compound ethinylestradiol (36). However, these genes were not upregulated in fish only exposed to the parasite (36). While, in the present study, the expression of *mcsf* occurred when almost every measured gene was upregulated before the molecule was downregulated at week 10 P.E at which time many transcripts were also downregulated, thus the regulation of this gene may be more indicative of a global transcriptional shift in immune response linked to the parasite. Furthermore, despite strong upregulation of *mcsf* no change in myeloid cells occurred in the AK at week 8 P.E. Hence, the biological consequences of this mRNA elevation or the mRNA levels of myeloid associated markers at any time point is difficult to disentangle from our data and does not directly correlate with pattern seen in levels of myeloid cells. A possible reason being the choice of genes measured.

In the present study *il-10*, a pleiotropic immunoregulatory molecule that can suppress innate immune responses; promote T cell proliferation and IgM production was one of the only transcripts significantly elevated at all time points in the PK in comparison to the control (37, 38). *il-10* has been found to be hyper expressed in several myxozoan infections of fish (39–41). In some of these studies it has been debated if this gene is induced by the immune system or the parasite. While we cannot deduce this from our study, our results advocated that strong *il-10* production in the PK is a consequence of high pathogen burdens as the expression level of the gene reduced with burden. Over production of *il-10* is associated with enhanced immunopathology and immunosuppression in response to several parasitic diseases in humans as well as an increased risk factor for development of autoimmune disease (42). This statement may apply to PKD given the immunopathology, reported immunosuppression (10, 43) and almost autoimmune like disease state induced in infected fish characterized by the massive lymphocyte proliferation, abnormal expression of Igs and *baff* transcripts that are all also common occurrences in autoimmune diseases of humans (33, 44). Excessive *il-10* is also considered a critical biomarker for poor disease outcome after infection in several parasitic diseases of mammals. On the other hand, abrogation of *il-10* signaling has also been alluded to enhanced survival (28, 45–47). This perspective might also be applied to *T. bryosalmonae* infection: in that moderate controlled mRNA levels of *il-10* indicate recovery, but overproduction is consistent with clinical infection as reported in earlier PKD studies (8, 10).

## Conclusion

In the right environmental conditions rainbow trout may recover from PKD (15), nevertheless, only limited information was known concerning the immune response that occurred during this disease phase (36). Our temporal analysis of rainbow trout commencing with the decline of parasitic burden during chronic PKD pathogenesis allowed an in-depth recognition of the dynamics and the interdependences between expressions of key B cell mechanisms in fish that were progressing to recovery. Although at a cellular level our temporal analysis would have benefitted from an increased number of samples. It could also be deemed a limitation of our study that we did not focus on elucidating tissue resolution mechanisms, but this was not the goal of the present study. Besides owing to the number of gene candidates potentially involved in tissue regeneration processes a sequencing approach would have been required and this was already performed by Bailey et al. (36) at a late stage of infection who found a low intensity immune response in *T. bryosalmonae* infected fish but did report on the regulation of many genes involved in tissue resolution and apoptosis.

While gaps still exist concerning the full trajectory in the immune response of rainbow trout infected with *T. bryosalmonae* particularly those which occur immediately after the host encounters the parasite. The knowledge generated here can be used to pinpoint if a fish is in recovery, this could be particularly important for studying an infected population in which no information is known about the initial infection timing or for investigating the impact of immunotherapies on the disease trajectory. Especially as tissue inflammation remains for a long period even after parasite clearance its usefulness as a proxy of recovery is not always informative enough compared to knowledge generated at the protein or cellular level. In summary, our approach of focusing on disease recovery as an alternative to disease development illuminate's novel information on the pathways that contribute to the re-establishment of host homeostasis, or recovery, after chronic infection by a myxozoan parasite. Future studies could also consider comparing these recovery processes to that in brown trout, which is not a dead-end host of the parasite.

## Data Availability Statement

The raw datasets supporting the conclusions of this article will be made available by the authors on request.

## Ethics Statement

The animal study was reviewed and approved by Cantonal veterinary office (Bern, Switzerland) (Authorization number BE60/14).

## Author Contributions

CB, CT, HS, and TW designed the experiments. CB collected the samples, performed the experiments, analyzed the data under the supervision of CT. CB and CT wrote the main body of the manuscript. CB complied figures/tables. All authors edited, read, and approved the final manuscript.

## Conflict of Interest

The authors declare that the research was conducted in the absence of any commercial or financial relationships that could be construed as a potential conflict of interest.
