# Supplementary material for: Back From the Brink: Alterations in B and T Cell Responses Modulate Recovery of Rainbow Trout From Chronic Immunopathological Tetracapsuloides bryosalmonae Infection
Source: Front Immunol. 2020 Jun 3;11:1093. doi: 10.3389/fimmu.2020.01093 (PMC7283781; doi:10.3389/fimmu.2020.01093)
Supplement: Supplementary file 1 [file Table_1.DOCX]

Supplementary Material

**Table. S1**. Sequences and corresponding accession numbers of rainbow trout primers used in this study

| Gene | Acc. number | FWD primer (5’-3’) | REV primer (5’-3’) | Ref. |
| --- | --- | --- | --- | --- |
| *ef1α* | AF498320 | TGCCCCTGGACACAGAGATT | CCCACACCACCAGCAACAA | (1) |
| *mcsf* | AM901600 | ACCCCGTCTGCCACGAATGA | CAGCTTGGCCCCAGCAACAG | (2) |
| *mcsf-r* | XM_031798057.1 | GTGAAGGGCAATGCGCGTC | CTGCTGGGACGGGAGAGTAGAAC | Here |
| *il-1β* | NM_001124347.2 | AGTGCTGTGGAAGAACATATAGTGTTG | CATCAGGACCCAGCACTTG | (3) |
| *blimp1* | XM_021600044.1 | AGCTGTCCAACCTCAAGGTCC | TTGCGGCACACCTGGGCATTC | (4) |
| *igt sec* | AY870268 | CATCAGCTTCACCAAAGGAAGTGA | TCACTTGTCTTCACATGAGTTACCCGT | (5) |
| *igt mem* | AY870264 | TCGAAGTCCACGGCGAACA | GTGTTCTTCACCGCTTCATCTTGAA | (5) |
| *igm sec* | X65261 | TACAAGAGGGAGACCGGAGGAGT | CTTCCTGATTGAATCTGGCTAGTGGT | (2) |
| *igm mem*  *igd sec*  *igd mem*  *cd38*  *baff* | OMU04616  JQ003979  AY870260  XM_021562832.1  NM_001124564.1 | CCTACAAGAGGGAGACCGATTGTC  TGGCACGCCAGGATTTGAC  CAGGAGGAAAGTTCGGCATCA  TCAAGTGAAACAACTGTCAAAAGA  GGTCACCTCCTCGTCTCTCT | GTCTTCATTTCACCTTGATGGCAGT  TCAGAATTGAGTGAACGGACAGACA  CCTCAAGGAGCTCTGGTTTGGA  ATAGGCCTCCATCGGTACATTACAT  TGTCTCTCCATGCCTAGCCT | (5)  (6)  (6)  Here  (7) |
| *pax5* | NM001124682 | ACGGAGATCGGATGTTCCTCTG | GATGCCGCGCTGTAGTAGTAC | (8) |
| *cd4* | AY973028 | CAACAGGGGCTGAAGATGTG | GCGGGATATCAAAGGTTGTG | (3) |
| *cd8α* | AY973028 | CCACGACGACTACACCAATG | GGCCCAAACAATCAACTCAC | (3) |
| *cd8β* | AY563420.1 | TCGTCAGCTGTGAGTGTTCC | CAGCATTGTTGAGGCTGAGA | (3) |
| *tcrβ* | AF329700.1 | TCACCAGCAGACTGAGAGTCC | AAGCTGACAATGCAQGGTCAAT | Here |
| *il-10* | NM_001245099.1 | CTGCTGCTCCTTCGTAGAGG | CTCGTCATTAGCCTCGTAGTAGTCTC | (3) |
| *cd137* | XM_021566191.1 | CTGCTGGTTCTCCTGATGTCTGCT | GTACCATCAGTTGTGTAGGTCTCATTCC | Here |
| *t-bet* | FM863825 | GTTCTGCAGTCGCTTCATAAGTACC | CTATGAATTGGGTCTCTGGGAAGAC | (6) |
| *gata3* | FM863826 | CCCATCGGTGCTAAACGAACA | GCTGTGGTGCTGCATTGCTT | (6) |
| *foxp3a* | FM883710 | CCCAGAACCGAGGTGGAGTGT | TGACGGACAGCGTTCTTCCA | (6) |

**
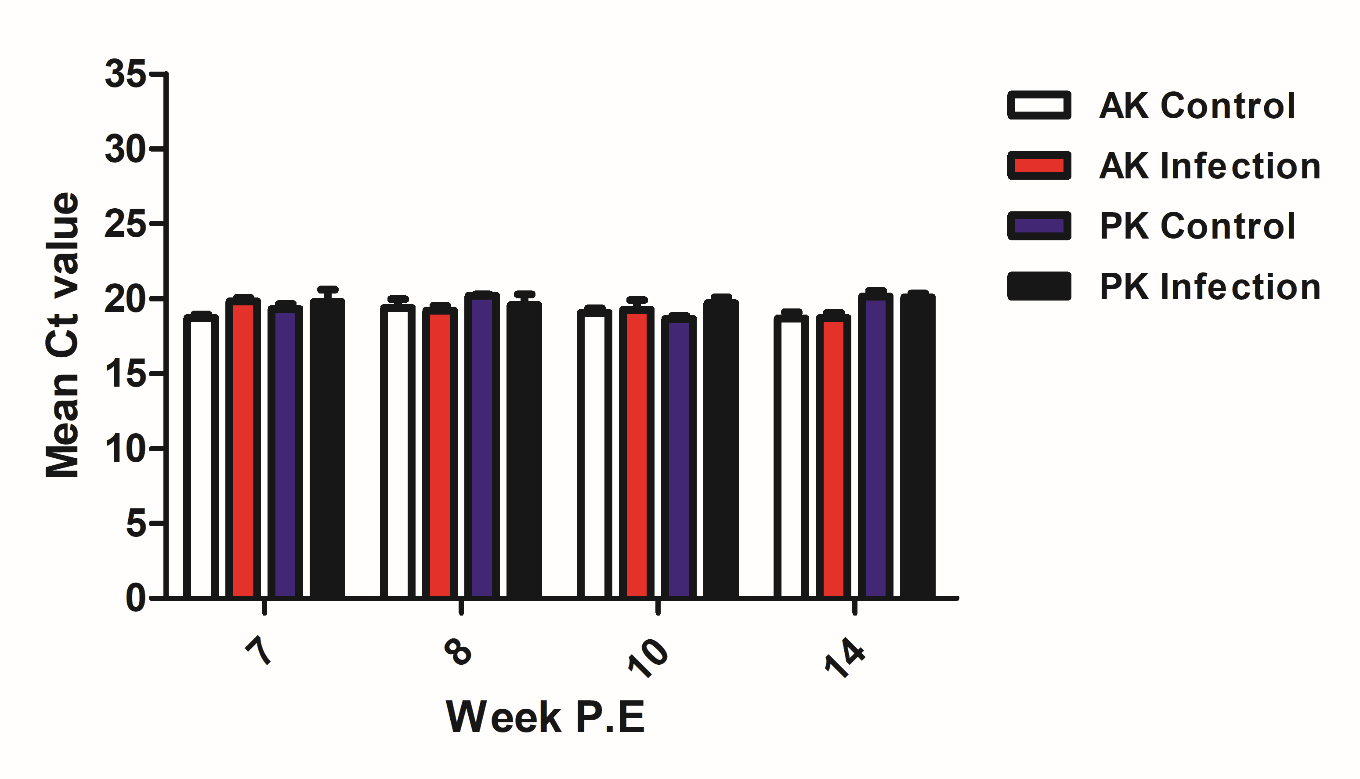
**

**Fig. S1.** Reference gene evaluation. The Ct values of the reference gene elongation factor 1α (*ef1α*) (Mean ± S.E) measured in control and *T. bryosalmonae* infected fish in the anterior kidney (AK) and the posterior kidney at day post-exposure (d.p.e). N=6 per time point per group per control and infection treatments.

**References**

1. C. Kropf, H. Segner, and K. Fent: ABC transporters and xenobiotic defense systems in early life stages of rainbow trout (Oncorhynchus mykiss). *Comp Biochem Phys C*. 185, 45-56 (2016) doi: 0.1016/j.cbpc.2016.02.006

2. B. Gorgoglione, W. Tiehui, C. J. Secombes and J. W. Holland: Immune gene expression profiling of Proliferative Kidney Disease in rainbow trout Oncorhynchus mykiss reveals a dominance of anti-inflammatory, antibody and T helper cell-like activities. *Vet Res*, 44((1):55) (2013) doi: 10.1186/1297-9716-44-55

3. C. Bailey, H. Segner, A. Casanova-Nakayama and T. Wahli: Who needs the hotspot? The effect of temperature on the fish host immune response to Tetracapsuloides bryosalmonae the causative agent of proliferative kidney disease. *Fish Shellfish Immunol*, 63, 424-437 (2017) doi: 10.1016/j.fsi.2017.02.039

4. P. Díaz-Rosales, S. Bird, T. Wang, K. Fujiki, W. Davidson, J. Zou and CJ Secombes: Rainbow trout interleukin-2: cloning, expression and bioactivity analysis. *Fish Shellfish Immunol*. 27(3), 414-22 (2009) doi.org: 10.1016/j.fsi.2009.06.008

5. A. Attaya, T. Wang, J. Zou, T. Herath, A. Adams and CJ Secombes and S. Yoon: Gene expression analysis of isolated salmonid GALT leucocytes in response to PAMPs and recombinant cytokines. *Fish Shellfish Immunol*, 80, 426-36 (2018) doi: 10.1016/j.fsi.2018.06.022

6. T. Wang, P. Johansson, B. Abós, A. Holt, C. Tafalla, Y. Jiang, A. Wang, Q. Xu, Z. Qi, W. Huang and MM. Costa: First in-depth analysis of the novel Th2-type cytokines in salmonid fish reveals distinct patterns of expression and modulation but overlapping bioactivities. *Oncotarge*t, 27, 109-117 (2016) doi: 10.18632/oncotarget.7295

7. C. Bailey, H. Segner and T. Wahli: What goes around comes around: an investigation of resistance to proliferative kidney disease in rainbow trout Oncorhynchus mykiss (Walbaum) following experimental re‐exposure. *J Fish Dis,* 40(11), 1599-1612 (2017) doi: 10.1111/jfd.12628

8. P. Zwollo, A. Haines, P. Rosato and J. Gumulak-Smith J. Molecular and cellular analysis of B-cell populations in the rainbow trout using Pax5 and immunoglobulin markers. *Dev Comp Immunol*, 32, 1482-1496 doi: 10.1016/j.dci.2008.06.008
